# Hypothyroidism attenuates protein tyrosine nitration, oxidative stress and renal damage induced by ischemia and reperfusion: effect unrelated to antioxidant enzymes activities

**DOI:** 10.1186/1471-2369-6-12

**Published:** 2005-11-07

**Authors:** Verónica M Tenorio-Velázquez, Diana Barrera, Martha Franco, Edilia Tapia, Rogelio Hernández-Pando, Omar Noel Medina-Campos, José Pedraza-Chaverri

**Affiliations:** 1 Facultad de Química, Departamento de Biología, Edificio B, Segundo Piso, Laboratorio 209, Universidad Nacional Autónoma de México (UNAM), Ciudad Universitaria, 04510, México, D.F., México; 2 Departamento de Nefrología, Instituto Nacional de Cardiología "Ignacio Chávez", Juan Badiano #1, Col. Sección XVI, 14080, Tlalpan, México, D.F., México; 3 Facultad de Medicina, Departamento de Farmacología, Universidad Nacional Autónoma de México (UNAM), Ciudad Universitaria, 04510, México, D.F., México; 4 Instituto Nacional de Ciencias Médicas y Nutrición "Salvador Zubirán", Departamento de Patología, 14000, México, D.F., México

## Abstract

**Background:**

It has been established that hypothyroidism protects rats against renal ischemia and reperfusion (IR) oxidative damage. However, it is not clear if hypothyroidism is able to prevent protein tyrosine nitration, an index of nitrosative stress, induced by IR or if antioxidant enzymes have involved in this protective effect. In this work it was explored if hypothyroidism is able to prevent the increase in nitrosative and oxidative stress induced by IR. In addition the activity of the antioxidant enzymes catalase, glutathione peroxidase, and superoxide dismutase was studied. Control and thyroidectomized (HTX) rats were studied 24 h of reperfusion after 60 min ischemia.

**Methods:**

Male Wistar rats weighing 380 ± 22 g were subjected to surgical thyroidectomy. Rats were studied 15 days after surgery. Euthyroid sham-operated rats were used as controls (CT). Both groups of rats underwent a right kidney nephrectomy and suffered a 60 min left renal ischemia with 24 h of reperfusion. Rats were divided in four groups: CT, HTX, IR and HTX+IR. Rats were sacrificed and samples of plasma and kidney were obtained. Blood urea nitrogen (BUN) and creatinine were measured in blood plasma. Kidney damage was evaluated by histological analysis. Oxidative stress was measured by immunohistochemical localization of protein carbonyls and 4-hydroxy-2-nonenal modified proteins. The protein carbonyl content was measured using antibodies against dinitrophenol (DNP)-modified proteins. Nitrosative stress was measured by immunohistochemical analysis of 3-nitrotyrosine modified proteins. The activity of the antioxidant enzymes catalase, glutathione peroxidase, and superoxide dismutase was measured by spectrophotometric methods. Multiple comparisons were performed with ANOVA followed by Bonferroni t test.

**Results:**

The histological damage and the rise in plasma creatinine and BUN induced by IR were significantly lower in HTX+IR group. The increase in protein carbonyls and in 3-nitrotyrosine and 4-hydroxy-2-nonenal modified proteins was prevented in HTX+IR group. IR-induced decrease in renal antioxidant enzymes was essentially not prevented by HTX in HTX+IR group.

**Conclusion:**

Hypothyroidism was able to prevent not only oxidative but also nitrosative stress induced by IR. In addition, the antioxidant enzymes catalase, glutathione peroxidase, and superoxide dismutase seem not to play a protective role in this experimental model.

## Background

Reactive oxygen species (ROS) [1,2] and reactive nitrogen species such as peroxynitrite (ONOO^-^) [3] are involved in the damage induced by ischemia and reperfusion (IR). The damage by reactive nitrogen species has been made evident by the increase in protein tyrosine nitration [3-6]. The consequences of IR include alterations in DNA, lipids, and proteins (carbonyl formation and nitrosylation) [3-6]. Renal IR is associated with acute renal failure [3,4] as well as proximal tubular damage [1-3]. IR-induced damage is ameliorated by spin traps [2], inhibition of inducible nitric oxide synthase [3], lecithinized superoxide dismutase (SOD) [3], ebselen, a ONOO^- ^scavenger [3], inhibitors of calpain activation [4], SOD and catalase (CAT) mimetic [6], antioxidants [7,8], or in the other circumstances such as hypothyroidism [9]. Paller [9] found that the renal damage and the increase in malondialdehyde (MDA) induced by IR were significantly lower in hypothyroid than in euthyroid rats. The specific mechanisms involved in the protective effect of hypothyroidism against renal IR remain to be fully elucidated.

The role of antioxidant enzymes in the oxidative damage to kidney has been studied. It has been found that the elevated expression of antioxidant enzymes including CAT, SOD, glutathione peroxidase (GPx) [10-15], and more recently heme oxygenase-1 [16,17], prior to renal oxidant insult, was able to ameliorate renal damage. Furthermore, the inhibition of CAT [18] or heme oxygenase-1 [16] aggravates renal damage induced by puromycin aminonucleoside [18] or IR [16], respectively. These data strongly suggest that the modulation of the antioxidant enzymes may alter the renal damage induced by oxidants. It is unknown if the antioxidant enzymes may be regulated differentially and involved in the protective effect of hypothyroidism against renal IR. Interestingly, the administration of some exogenous antioxidants is able to modulate antioxidants enzymes and renal damage induced by IR [19-21]. In addition, (-)-epicatechin 3-O-gallate [22] and Wen-Pi-Tang [23] induced renal antioxidant enzymes and protected against lipopolysaccharide- and IR-induced kidney damage and plasma 3-nitrotyrosine (3-NT) formation. Tyrosine nitration may be induced not only by ONOO^-^, but also by another reactive nitrogen species including nitrogen dioxide radical (NO_2_^•^) and dinitrogen trioxide (N_2_O_3_). ONOO^- ^is a potent oxidation species that have been found to cause also lipid peroxidation and cytotoxicity [24-26].

Based on the above information, in the present paper we evaluated if hypothyroidism is able to prevent against the nitrosative stress induced by IR. Nitrosative stress was evaluated by measuring nitrated proteins by immunohistochemistry using antibodies against 3-NT [3-6,27,28]. Oxidative stress was evaluated using immunohistochemical techniques to evaluate the protein carbonyl content [29] and 4-hydroxy-2-nonenal (4-HNE) protein adducts [30,31]. The protein carbonyl content was measured using antibodies against dinitrophenol (DNP)-modified proteins [29]. In addition the activity of the antioxidant enzymes CAT, GPx, and SOD was studied before and after renal IR in control and hypothyroid rats.

## Methods

### Reagents

Xanthine, nitroblue tetrazolium (NBT), 3,3-diaminobenzidine, bovine serum albumin, xanthine oxidase, NADPH, glutathione reductase (GR), 2,4-dinitrophenylhydrazine, and reduced glutathione (GSH) were purchased from Sigma (St. Louis, MO, USA). Ethylenediaminetetraacetic acid disodium salt (EDTA Na_2_), ammonium sulfate, and copper chloride were purchased from JT Baker (Mexico City, México). Hydrogen peroxide (H_2_O_2_), formaldehyde, and sodium carbonate were obtained from Mallinckrodt (Paris, KY, USA). Sodium azide was obtained from Merck (Mexico City, México). Rabbit anti-3-NT polyclonal antibodies were from Upstate (Catalogue # 06-284, Lake Placid, NY, USA). Goat anti-DNP polyclonal antibodies (Catalogue # J06) were from Biomeda Corporation (Foster City, CA, USA). Mouse anti-4-HNE monoclonal antibodies (Catalogue #24325) were from Oxis International Inc. (Portland, OR, USA). Anti-rabbit Ig horseradish peroxidase antibody (Catalogue # NA-934) and anti-mouse Ig horseradish peroxidase antibody (Catalogue # NIF-825) were purchased from Amersham Life Sciences (Buckinghamshire, England). Donkey anti-goat horseradish peroxidase antibodies (Catalogue # SC2020) were from Santa Cruz Biotechnology, Inc. (Santa Cruz, CA, USA). All other chemicals were reagent grade and commercially available.

### Induction of hypothyroidism

All animal procedures were approved by the Animal Care Committee of the Instituto Nacional de Cardiología "Ignacio Chavez" and followed the guidelines of Norma Oficial Mexicana (NOM-ECOL-087-1995). Male Wistar rats weighing 380 ± 17 g underwent surgical thyroidectomy with parathyroid reimplant (HTX), as previously described [32-35]. Briefly, the trachea was exposed under ether anesthesia, and under a stereoscopic microscope (Wild M5, Wild Heerbrugg, Switzerland), the parathyroid glands were visualized, dissected from the thyroid gland, and reimplanted into the surrounding neck muscles. The thyroid gland was then carefully dissected, to avoid injury to the laryngeal nerves, and completely excised. The effectiveness of this procedure was assessed by determining the concentration of calcium, phosphorous and thyroxine in 10 sham-operated control and 10 HTX rats, using standard techniques. The results obtained 15 days after surgery were: Ca^2+ ^of 10.2 ± 3 in control vs. 10.3 ± 0.2 mM in HTX; phosphorous of 6.5 ± 0.3 in control vs. 6.3 ± 0.5 mM in HTX; and thyroxine of 6.4 ± 0.77 in control vs. 1.18 ± 0.19 μg dL^-1 ^in HTX, *P *< 0.05. The sham group (375 ± 14 g) underwent a surgical procedure in which the animals were anesthetized, the trachea was exposed and then the incision was closed simulating the thyroidectomy surgery. The body weight for HTX and CT rats obtained 15 days after surgery was 389 ± 22 g and 417 ± 25 g, respectively.

### Ischemia and reperfusion studies

Four groups of rats were studied: Control (CT), sham operated animals; hypothyroid (HTX), rats subjected to thyroidectomy; ischemia and reperfusion (IR), rats submitted to IR; and HTX+IR, rats subjected to HTX plus IR. The experimental protocol was performed 15 days after the thyroidectomy (HTX group) or the simulated surgery (CT). Under anesthesia and heparin administration, blood samples were obtained and the kidneys were reperfused and removed. Additional animals from CT and HTX groups were subjected to right nephrectomy and the left renal artery was occluded with a non-traumatic vascular clamp for 60 min. Then, the clamp was released allowing the reestablishment of renal blood flow or reperfusion and 24 h after the rats were anesthetized, blood samples were obtained and the kidney was washed with 0.9% saline solution and excised. These groups were named as IR and HTX+IR, respectively. Blood plasma was obtained and stored at -40°C. Kidney was used for histological and immunohistochemical studies and for determination of antioxidant enzymes activities. Areas of the kidney (renal cortex, outer medulla and inner medulla) were macroscopically dissected using a razor blade and frozen at -70°C for further measurement of enzymatic activities.

### Determination of plasma creatinine and blood urea nitrogen (BUN)

Creatinine and BUN were measured using a creatinine analyzer model 2 and a BUN analyzer 2 (Beckman Instruments, Fullerton, CA, USA), respectively.

### Histological studies

Kidney tissue slices were fixed in 10% neutral buffered formaldehyde solution, and embedded in paraffin [27,36,37]. Sections at 4 μm of thickness were obtained and stained with hematoxylin-eosin (H&E). Histologic assessment of tubular necrosis was determined semiquantitatively using a method described by Chatterjee *et al*. [38]. The score was graded from 0 to 3 where 0 = normal histology, 1 = tubular cells swellings, brush border loss, nuclear condensation, with up to one third of tubular profile showing nuclear loss; 2 = same as for score, but greater than one third and less than two thirds of tubular profile shows nuclear loss; 3 = grater of two thirds of tubular profile shows nuclear loss. In addition a quantitative histological damage was determined by using a Leica Qwin Image Analyzer (Leica Microsystems, Cambridge, UK). The following parameters were quantified: (a) the percentage of tubules with hyaline casts in the renal cortex, using the low power magnification objective, three random choice fields were studied counting the tubules without and with hyaline casts to determine the percentage of the latter, and (b) in those tubules with hyaline casts, the total lumen area and the area occupied by the cast were determined, then the percentage of the lumen area occupied by the hyaline cast was determined.

### Immunohistochemical localization of 3-NT, DNP, and 4-HNE

For immunohistochemistry, 4 μm sections were deparaffinized with xylene and rehydrated with ethanol. Endogenous peroxidase was quenched/inhibited with 4.5% H_2_O_2 _in methanol by incubation for 1.5 h at room temperature. The sections used for DNP immunohistochemistry were incubated with 0.2% dinitrophenylhydrazine in 2 N HCl for 60 min at room temperature in absence of light and then were extensively washed. Nonspecific adsorption was minimized by leaving the sections in 3% bovine serum albumin in phosphate buffer saline for 30 min. Sections were incubated overnight with 1:700 dilution of anti-3-NT antibody [36] or with 1:500 dilution of anti-DNP antibody or with 1:100 dilution of anti-4-HNE antibody [36]. After extensive washing with phosphate buffer saline, the sections were incubated with 1:500 dilution of peroxidase conjugated anti-rabbit Ig antibody (for 3-NT) or with a 1:500 dilution of a peroxidase conjugated anti-goat Ig or anti-mouse IgG (for DNP and 4-HNE, respectively) for 1 h, and finally incubated with H_2_O_2_-diaminobenzidine for 1 min. Sections were counterstained with hematoxylin (for 3-NT and 4-HNE) or with methyl green (for DNP) and observed under light microscopy. All the sections from the four studied groups were incubated under the same conditions with the same antibodies concentration, and in the same running, so the immunostaining was comparable among the different experimental groups. Quantitative image analysis was performed with a Zeiss KS 300 Imaging System 3.0 (Carl Zeiss Vision GmbH, Hallbergmoos, Germany). This software determines densitometric means value of selected tissue regions. Thus, 10 fields/rat were randomly selected and the intensity of the 3-NT, 4-HNE, and DNP immunostaining was determined. We normalized the data (arbitrary units) to 1.0 using the control kidneys. We run negative controls omitting primary and/or secondary antibodies.

### Tissue homogenization

Renal cortex, outer medulla and inner medulla were homogenized in a Polytron (Model PT 2000, Brinkmann, Westbury, NY, USA) for 10 seconds in cold 50 mM potassium phosphate, 0.1% Triton X-100, pH = 7.0 [28]. The homogenate was centrifuged at 19,000 × g and 4°C for 30 min and the supernatant was separated to measure total protein and the activities of CAT, GPx, and SOD. Total protein was measured by the method of Lowry *et al*. [39].

### Catalase assay

Renal CAT activity was assayed at 25°C by a method based on the disappearance of H_2_O_2 _from a solution containing 30 mM H_2_O_2 _in 10 mM potassium phosphate buffer pH 7.0 at 240 nm [40]. The reaction was started by the addition of 25 μL of the sample to 725 μL of H_2_O_2_. Under the described conditions, the decomposition of H_2_O_2 _by CAT contained in the samples follows a first-order kinetic as given by the equation *k *= 2.3/*t *log *Ao/A *where *k *is the first-order reaction rate constant, *t *is the time over which the decrease of H_2_O_2_, due to CAT activity, was measured (15 s), and *Ao/A *is the optical density at times 0 and 15 s, respectively. The results were expressed in *k*/mg protein.

### Glutathione peroxidase assay

Renal GPx activity was assayed by a method previously described [41]. Reaction mixture consisted of 50 mM potassium phosphate pH = 7.0, 1 mM EDTA, 1 mM sodium azide, 0.2 mM NADPH, 1 U/mL of glutathione reductase, and 1 mM GSH. One hundred μL of the appropriate dilution of tissue homogenates were added to 0.8 mL of mixture and allowed to incubate for 5 min at room temperature before initiation of the reaction by the addition of 0.1 mL 2.5 mM H_2_O_2 _solution. Absorbance at 340 nm was recorded for 3 min and the activity was calculated from the slope of these lines as μmoles of NADPH oxidized per min taking into account that the millimolar absorption coefficient for NADPH is 6.22 mM^-1^cm^-1^. Blank reactions with homogenates replaced by distilled water were subtracted from each assay. The results were expressed as U/mg protein.

### Superoxide dismutase assay

SOD activity in kidney homogenates was assayed by using a previously reported method [41]. A competitive inhibition assay was performed using xanthine-xanthine oxidase system to reduce NBT. Mixture reaction contains in a final concentration: 0.122 mM EDTA, 30.6 μM NBT, 0.122 mM xanthine, 0.006% bovine serum albumin, and 49 mM sodium carbonate. Five hundred μL of tissue homogenate at the appropriate dilution, were added to 1.66 mL of the mixture described above, then 50 μL xanthine oxidase, in a final concentration of 2.8 U/L, were added and incubated in a water bath at 27°C for 30 min. The reaction was stopped with 066 mL of 0.8 mM cupric chloride and the optical density was read at 560 nm. One hundred percent of NBT reduction was obtained in a tube in which the sample was replaced by distilled water. The amount of protein that inhibited NBT reduction to 50% of maximum was defined as one unit of SOD activity. Results were expressed as U/mg protein.

### Statistics

The data are expressed as the mean ± SD. Data were analyzed with a non-paired t-test or with ANOVA followed by multiple comparisons by Bonferroni t test, as appropriate. P value less than 0.05 was considered statistically significant.

## Results

### General and biochemical data

Creatinine and BUN were similar in CT and HTX groups (Table 1). Twenty four h after IR, both plasma creatinine and BUN increased significantly, however the increases were significantly lower in HTX+IR group (Table 1). These data confirm previous observations of Paller [9] who showed that the renal damage induced by IR was ameliorated in HTX rats.

### Histological and immunohistochemical analysis

**Figure 1:**
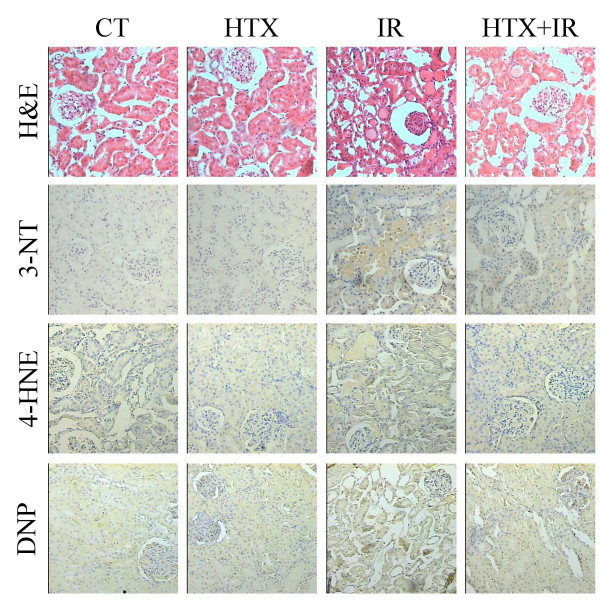
Representative images of histology (H&E) (first row) and immunohistochemical detection of 3-NT (second row), 4-HNE (third row) and DNP (fourth row) in the renal cortex of the four groups of rats studied: Control (CT), thyroidectomized (HTX), ischemia and reperfusion (IR) and HTX+IR (100× magnification).

**Figure 2:**
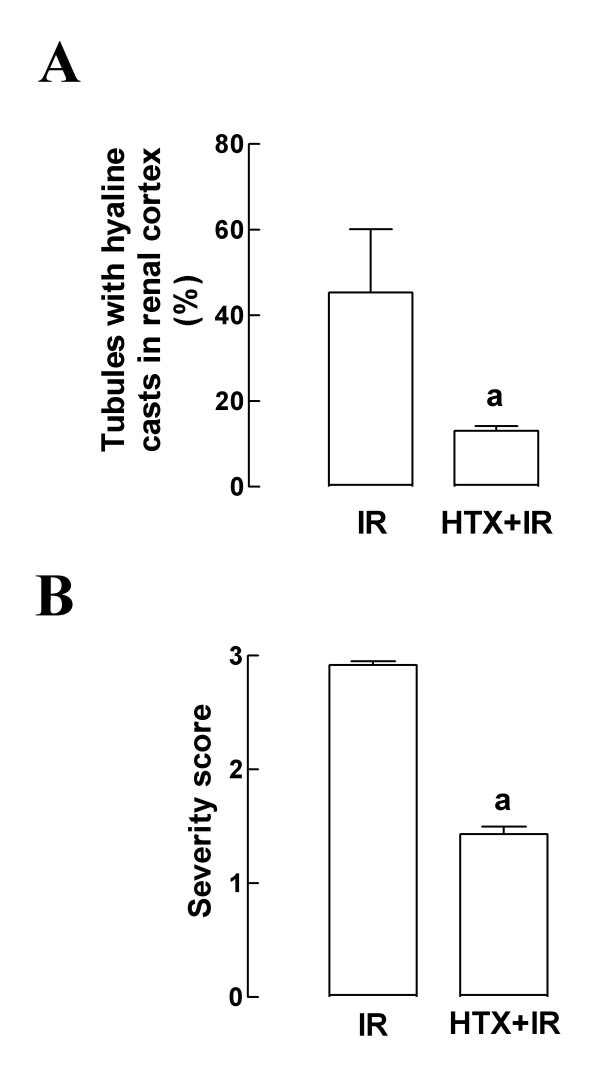
Quantitative analysis of damage in the renal cortex of rats from IR and HTX+IR groups. (A) Percentage of tubules with hyaline casts. n = 4 rats and 60 tubuli/rat. (B). Severity score. Mean ± SD. n = 4 rats and 15 tubuli/rat. ^a^P < 0.001 vs. IR.

Representative histopathology and immunohistochemistry features in the kidney cortex of rats after 24 h of IR in control and HTX rats are presented in Figure 1. Surgical induced hypothyroidism does not produce any histological abnormality in the kidney. After IR, the kidney cortex shows extense ischemic tubular necrosis and hyaline cylinders are present in many tubular lumens (Figures 1 and 2).

**Figure 3:**
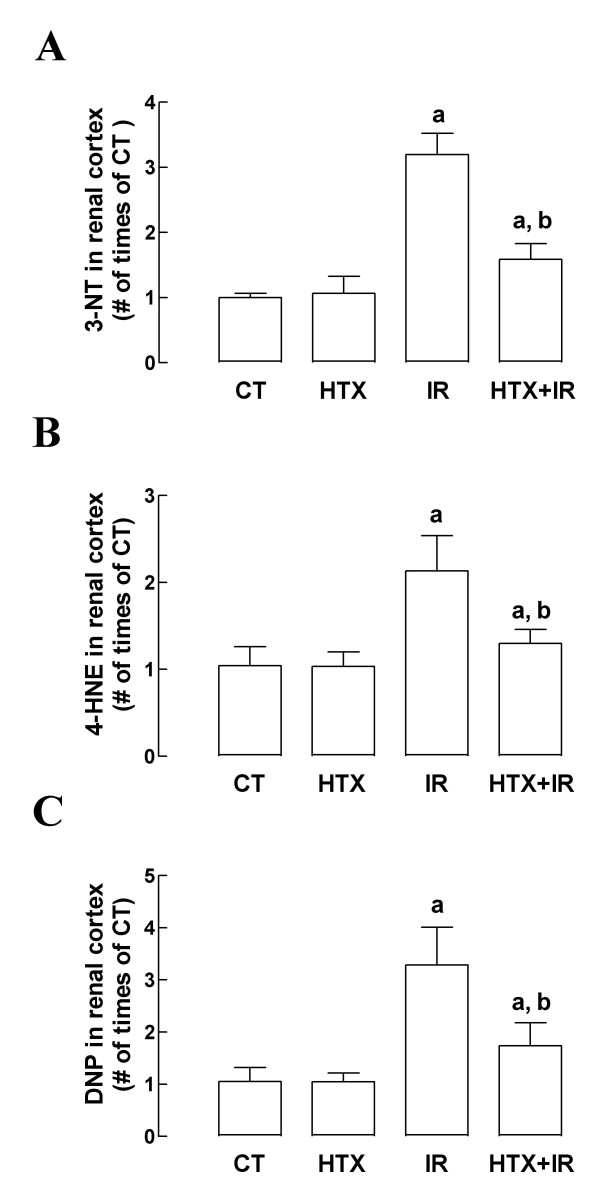
Quantitative immunostaining of 3-NT, 4-HNE, and DNP-modified proteins in renal cortex of the four groups of rats studied: CT, HTX, IR, and HTX+IR. n = 4 rats and 10 determinations/rat. Mean ± SD. ^a^P < 0.001 vs. control, ^b^P < 0.001 vs. IR.

The histological abnormalities, manifested by numerous necrotic tubules with a high percentage of tubules with hyaline casts, were significantly lower in the HTX+IR group (HTX+IR group vs. IR group P < 0.001, Figure 2) confirming that hypothyroidism prevented renal damage induced by IR (Figure 1). In a similar fashion than in CT rats, the immunostaining of 3-NT, 4-HNE, and DNP is negative in HTX rats, while it is strong in the IR group (Figure 3). The increase in 3-NT, 4-HNE, and DNP staining induced by IR was ameliorated in the HTX+IR group (Figures 1 and 3).

**Figure 4:**
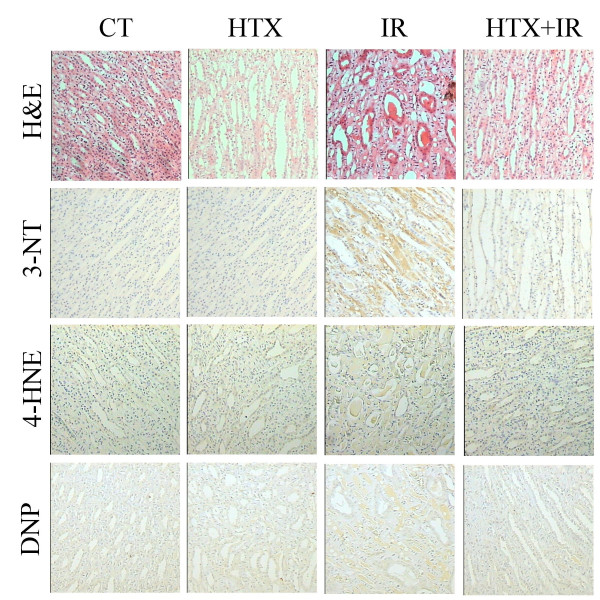
Representative images of histology (H&E) (first row) and immunohistochemical detection of 3-NT (second row), 4-HNE (third row) and DNP (fourth row) in renal medulla of the four groups of rats studied: CT, HTX, IR, and HTX+IR. (100× magnification).

**Figure 5:**
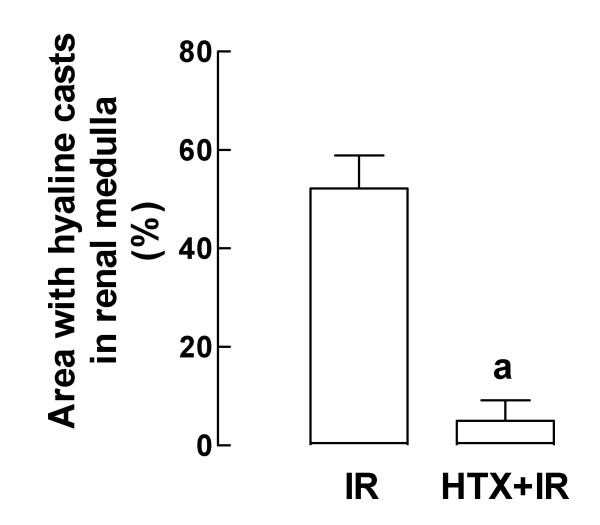
Quantitative analysis of damage in the renal medulla of rats from IR and HTX+IR groups. Area of tubular lumen with hyaline casts is shown as percentage. n = 4 rats, and 60 tubular lumen/rat. Mean ± SD. ^a^P < 0.001 vs. IR.

**Figure 6:**
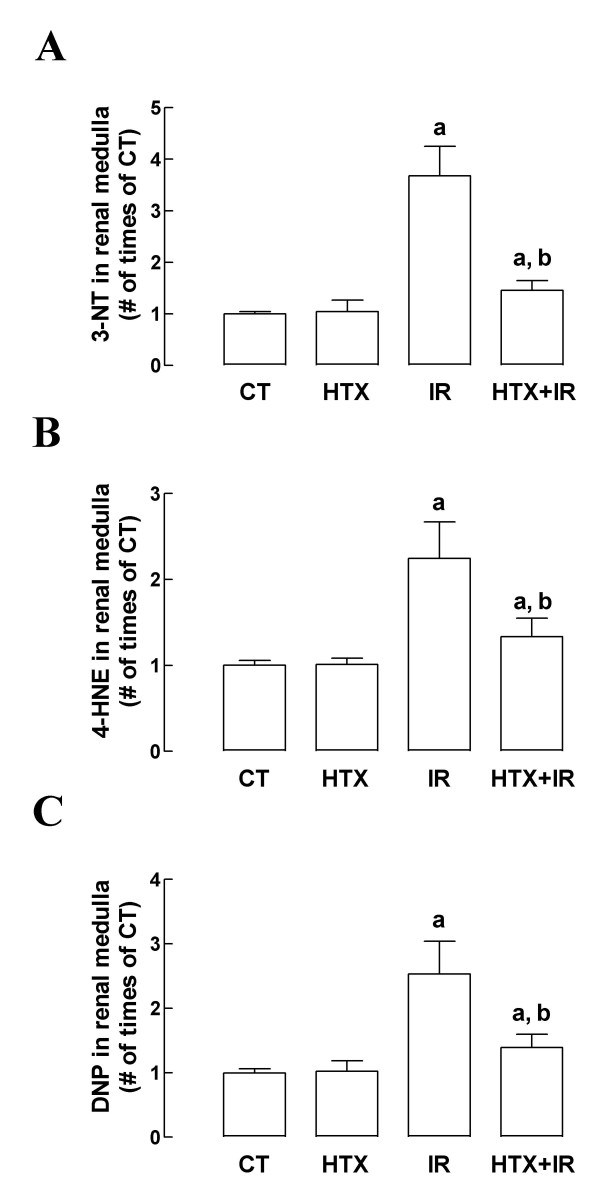
Quantitative immunostaining of 3-NT, 4-HNE, and DNP-modified proteins in renal medulla of the four groups of rats studied: CT, HTX, IR, and HTX+IR. n = 4 rats and 10 determinations/rat. Mean ± SD. ^a^P < 0.001 vs. control, ^b^P < 0.001 vs. IR.

Representative histopathology and immunohistochemistry features in the medulla of the kidney rats in the four groups of animals is presented in Figure 4. There are no abnormalities in the renal medulla in HTX group. After IR, the kidney medulla shows extense ischemic tubular necrosis, and numerous tubular lumens have hyaline casts (Figure 4). Ischemia and reperfusion in HTX rats produced lesser histological damage, occasional medullar kidney tubules are revisted by necrotic epithelium (Figure 4) and a decrease in the area of tubular lumen occupied with hyaline casts (HTX+IR group vs. IR group, P < 0.001, Figure 5). Like in control rats, the immunostaining of 3-NT, 4-HNE and DNP is negative in HTX rats, whereas it is strong after IR (Figure 4). The staining of 3-NT, HNE and DNP in HTX was significantly lower in HTX+IR group compared to IR group (Figures 4 and 6).

### Renal activity of antioxidant enzymes

The renal activity of antioxidant enzymes in CT and HTX rats before and after IR is shown in tables 2, 3, 4. Activities of CAT (Table 2) and GPx (Table 3) were measured in renal cortex and outer and inner medulla. Superoxide dismutase was measured in renal cortex and outer medulla (Table 4). There was no enough sample of inner medulla to measure SOD. With one exception (a marginal increase in GPx activity in outer medulla), the comparisons between CT and HTX rats were not significant. Ischemia and reperfusion induced a decrease in the antioxidant enzymes. Catalase activity was decreased in renal cortex of IR group compared to CT group and in renal cortex and outer medulla of HTX+IR group compared to HTX group (Table 2). Glutathione peroxidase activity decreased in renal cortex and outer medulla of IR group compared to CT group and in renal cortex and inner medulla of HTX+IR group compared to HTX group (Table 3). Superoxide dismutase activity decreased in outer medulla of HTX+IR group compared to HTX group (Table 4). With one exception (GPx in outer medulla) the IR-induced decrease in renal antioxidant enzymes was not prevented by HTX in HTX+IR group. In fact, the activities of antioxidant enzymes were significantly lower in HTX+IR group than in IR group. This may suggest that the antioxidant enzymes in outer medulla HTX group are more susceptible to inactivation by IR than those of CT rats.

### Discussion

The data presented in this work show that hypothyroid rats were significantly more resistant to IR induced renal damage than euthyroid rats and are consistent with the protective effect of hypothyroidism against oxidative stress and tissue damage in several experimental models [42-44]. The protective effect of HTX was observed by histological (necrotic tubules, percentage of tubules with hyaline casts and area of tubular lumen occupied by hyaline casts) and biochemical (creatinine and BUN) analyses. In addition, this protective effect was associated with a decrease in oxidative damage which was evaluated by the immunohistochemical localization of protein carbonyls and 4-HNE modified proteins. The protective effect of HTX in ischemia and reperfusion associated with the amelioration of oxidative damage had been observed previously by Paller [9]. Although hypothyroidism protects the kidney during IR, the mechanism involved in this protective effect has not been completely elucidated. One of the major effects of thyroid hormones is to increase mitochondrial respiration [45] which results in increased generation of ROS, leading to oxidative damage to membrane lipids. There is a good deal of evidence to indicate that metabolic depression brought about by hypothyroidism is associated with a decrease in free radical production and a subsequent protection against lipid peroxidation [46,47]. This supports the notion that reduced demand for oxygen in hypothyroidism serves as a protective factor in tissue injury due to ROS. In fact, it has been shown that hypothyroidism is able to prevent the increase in lipid peroxidation and the diminution in GSH as well the tissue damage induced by intracolonic administration of trinitrobenzene sulfonic acid (experimental model of colitis) [42]. Hypothyroidism also prevents the increase in MDA and decreases the susceptibility to oxygen radical-induced lung damage in newborn rats exposed to prolonged hyperoxia [43]. The lower toxicity of arsenic in hypothyroid animals was associated with the prevention of arsenic-induced lipid peroxidation in liver and kidneys [44]. Furthermore, hypothyroidism was able to protect against acetaminophen hepatotoxicity [48] which has been associated to oxidative stress [49]. The majority of the studies in hypothyroid animals have found no change or a decrease in tissue markers of oxidative stress (thiobarbituric acid reactive substances, MDA or oxidized glutathione) (Table 5) supporting a decreased production of ROS in this experimental model.

In this study we also showed that IR damage was associated with an increase in tyrosine nitration which is consistent with previous studies [3-6]. Interestingly, it was observed that the protective effect of HTX was associated with a significant decrease in 3-NT immunostaining. Noiri *et al*. [3], Chatterjee *et al*. [4,6], and Patel *et al*. [5] found that the protective effect of ebselen [3], PD150606 and E-64 (inhibitors of calpain activation) [4], interleukin-6 deficiency [5], and EUK-134 (a SOD and CAT mimetic) [6] in renal ischemia and reperfusion damage was associated with attenuation of nitrosative stress evaluated by tyrosine nitration. More recently it was shown that the protective effect of soy feeding of renal damage induced by puromycin aminonucleoside was associated with a decrease in tyrosine nitration [58].

Tyrosine nitration is induced by reactive nitrogen species including ONOO^- ^which is synthesized by the reaction between superoxide anion (O_2_^•↓^) and nitric oxide (NO^•^). The protective effect of ebselen, a ONOO^- ^scavenger, in the IR-induced renal damage and tyrosine nitration suggests that ONOO^- ^is enhanced and involved in tissue damage and nitrosative stress in this experimental model. There are evidences suggesting the increase of O_2_^•↓ ^and NO^• ^in IR-induced renal damage [reviewed in [59]] which may favor ONOO^- ^formation. In hypothyroid rats, the decreased nitrosative stress may be explained, at least in part, by the diminution in oxygen consumption and O_2_^•↓ ^production.

We wanted to know if the antioxidant enzymes may be involved in the protective effect of HTX in IR taking into account several reports of the literature showing that the enhanced expression of some antioxidant enzymes was able to attenuate renal damage induced by IR [11,16], H_2_O_2 _infusion [10,11], cisplatin [13], puromycin aminonucleoside [10], and cyclosporine A [15]. However, our data show that IR induced a decrease in the activity of CAT, GPx and SOD. Our data are consistent with previous data showing that renal ischemia and reperfusion damage is associated with a decrease in antioxidant enzymes [19,20,60]. Interestingly, the IR-induced decrease in antioxidant enzymes was not prevented by HTX in the HTX+IR group. In fact, in some cases, the IR-induced decrease in antioxidant enzymes was significantly higher in the HTX+IR group compared to IR group suggesting that these antioxidant enzymes are not involved in the protective effect of HTX on IR-induced renal damage and oxidative and nitrosative stress. Antioxidant enzymes activities in CT and HTX groups were essentially similar suggesting that hypothyroidism has no effect on the renal activity of these enzymes. There is no a consistent pattern on the effect of hypothyroidism on tissue antioxidant enzymes (see Table 6). Sawant *et al*. [67] found that SOD decreased, GPx increased and CAT remained unchanged in hypothyroid rats.

The most studied enzymes in hypothyroid animals are SOD, Cu, ZnSOD, MnSOD, CAT, GPx, and GR. The effect of hypothyroidism on the antioxidant enzymes in several tissues is not consistent (Table 6). In some cases the change of antioxidant enzyme activity seems to be tissue specific [47,68]. On the other hand, within a single tissue, the response of the antioxidant enzymes to hypothyroidism is not always similar [55,61-67].

Hypothyroidism attenuates not only renal but also cardiac damage induced by ischemia and reperfusion. Bobadilla *et al*. [69] have shown that hypothyroidism conferred protection against reperfusion arrhythmias and the cardiac release of creatine kinase and aspartate amino transferase and preserved the normal structure of myocardial tissue. In addition Chavez *et al*. [70] demonstrated that hypothyroidism renders mitochondria resistant to the opening of membrane permeability transition pore. This may be relevant to the protective effect of hypothyroidism in ischemia and reperfusion since it has been recognized that mitochondria play a key role in cell-death pathways by activating mitochondrial permeability transition pore and causing the release of cytochrome C and proapoptotic factors, as well as Ca^2+ ^overload that promotes non-selective permeability of the inner membrane. The prolonged opening of the membrane permeability transition pore during the first few minutes of reperfusion is a critical determinant of cell death, and pharmacological inhibition of the pore at the time of reperfusion protects the cells [71].

## Conclusion

It is concluded that HTX rats are more resistant to oxidative and nitrosative stress and renal damage induced by IR, which is not mediated by a differential regulation of the antioxidant enzymes CAT, GPx, and SOD.

## List of abbreviations used

ANOVA Analysis of variance

BUN Blood urea nitrogen

CAT Catalase

CT Control rats

DNP Dinitrophenol

GPx Glutathione peroxidase

GR Glutathione reductase

GSH Glutathione, reduced form

GSSG Glutathione, oxidized form

4-HNE 4-hydroxy-2-nonenal

H&E Hematoxylin-eosin

HTX Hypothyroid rats

IR Ischemia and reperfusion

MDA Malondialdehyde

MMI Methimazole

3-NT 3-nitrotyrosine

NBT Nitroblue tetrazolium

ONOO^-^Peroxynitrite

PTU 6-n-propyl-2-thiouracil,

ROS Reactive oxygen species

SD Standard deviation

SOD Superoxide dismutase

## Competing interests

The author(s) declare that they have no competing interests.

## Authors' contributions

VMTV performed ischemia and reperfusion studies, collected samples and measured the activity of antioxidant enzymes. DB performed histological and immunohistochemical analyses and edited the manuscript. MF thyroidectomized rats and characterized the hypothyroid state, performed ischemia and reperfusion studies and edited the manuscript. ET performed ischemia and reperfusion studies and edited the manuscript. RHP advised about the histological and immunohistochemical analyses. ONMC performed the statistical analyses and edited the manuscript. JPCH conceived and coordinated the study and wrote and edited the manuscript.

## Pre-publication history

The pre-publication history for this paper can be accessed here:



## Figures and Tables

**Table 1:** Plasma creatinine and BUN in the four groups of rats studied.

	CT	HTX	IR	HTX+IR
Creatinine, mg/dL	0.45 ± 0.05	0.40 ± 0.08	5.08 ± 0.55^a^	3.83 ± 0.41*
BUN, mg/dL	26 ± 2	23 ± 1	122 ± 11^a^	78 ± 17*

Data are mean ± SD. N = 16. ^a^P < 0.001 vs. CT, *P < 0.001 vs. IR.

**Table 2:** Catalase activity (*k*/mg protein) in the four groups of rats studied.

	CT	HTX	IR	HTX+IR
Renal cortex	0.22 ± 0.04 (16)	0.22 ± 0.02 (16)	0.17 ± 0.05^a ^(5)	0.14 ± 0.04^b ^(5)
Outer medulla	0.07 ± 0.02 (16)	0.08 ± 0.02 (16)	0.08 ± 0.017 (7)	0.04 ± 0.004^b,* ^(7)
Inner medulla	0.013 ± 0.002 (16)	0.014 ± 0.003 (16)	0.012 ± 0.003 (7)	0.012 ± 0.002 (7)

Data are mean ± SD. Number of determinations is in parentheses. ^a^P < 0.05 vs. CT, ^b^P < 0.001 vs. HTX, *P < 0.01 vs IR.

**Table 3:** Glutathione peroxidase activity (U/mg protein) in three sections of kidney from the four groups of rats studied.

	CT	HTX	IR	HTX+IR
Renal cortex	0.10 ± 0.01 (16)	0.11 ± 0.01 (16)	0.07 ± 0.03^a ^(7)	0.04 ± 0.01^b,** ^(7)
Outer medulla	0.07 ± 0.01 (16)	0.06 ± 0.01* (16)	0.03 ± 0.003^a ^(7)	0.05 ± 0.007*** (7)
Inner medulla	0.06 ± 0.02 (16)	0.05 ± 0.01 (16)	0.05 ± 0.02 (6)	0.03 ± 0.003 (6)

Data are mean ± SD. Number of determinations is in parentheses. ^a^P < 0.001 vs. CT, ^b^P < 0.001 vs. HTX, *P < 0.05, vs. CT, **P < 0.01, ***P < 0.001 vs. IR.

**Table 4:** Superoxide dismutase activity (U/mg protein) in the four groups of rats studied.

	CT	HTX	IR	HTX+IR
Renal cortex	13.3 ± 1.0 (16)	14.4 ± 1.0 (16)	15.1 ± 2.1 (7)	13.4 ± 3.2 (7)
Outer medulla	10.9 ± 1.3 (16)	10.4 ± 1.1 (16)	8.2 ± 1.2^a ^(8)	5.6 ± 1.5^b,* ^(8)

Data are mean ± SD. Number of determinations is in parentheses. ^a^P < 0.001 vs. CT, ^b^P < 0.001 vs. HTX, *P < 0.001 vs. IR.

**Table 5:** Effect of experimental hypothyroidism on oxidative stress markers.

Ref.	Specie	MHI	Change in oxidative stress markers
9	Rat	HTX	↔ MDA and ↑ GSH in renal cortex.
50	Rat	PTU	↑ Brain total antioxidant status.
51	Rat	PTU	↓ MDA and GSH levels in cerebral, hepatic and cardiac tissues.
52	Rat	PTU	↓ Advanced glycation end-products and MDA-lysine in liver.
53	Rat	HTX	↑ GSH and ↓ MDA levels in liver.
54	Rat	PTU	↑ MDA in plasma, erythrocytes, and liver tissue. ↔ MDA in kidney. ↔ GSH levels of kidney and liver.
55	Rat	MMI	↔ Brain TBARS.
56	Mouse	PTU	↔ LPx and GSH, GSSG and GSSG/GSH ratio in skeletal muscle.
47	Rat	PTU	↓ TBARS in extensor digitorum longus muscle. ↔ TBARS in heart, liver, and soleus muscle.
57	Rat	PTU	↓ MDA and GSH in renal and testicular tissue.

Ref. = reference, MHI = Method of hypothyroidism induction, HTX = Thyroidectomized, PTU = 6-n-propyl-2-thiouracil, MMI = Methimazole, ↑ = increase, ↓ = decrease, ↔ = without change, LPx = lipid peroxidation, MDA = malondialdehyde, GSH = reduced glutathione, GSSG = oxidized glutathione, TBARS = Thiobarbituric acid reactive substances.

**Table 6:** Effect of experimental hypothyroidism on antioxidant enzymes activities.

Ref.	Specie	MHI	Change in antioxidant enzymes
61	Rat	MMI	↑ MnSOD and CAT and ↔ Cu, ZnSOD in brown adipose tissue.
62	Rat	PTU	In liver mitochondria: ↑ Total and Cu, ZnSOD, ↔ MnSOD, ↓ CAT. ↑ Total and Se-independent and Se-dependent GPx
63	Rat	PTU	↓ SOD and ↔ CAT in heart.
64	Rat	MMI	↑ GPx, ↔ CAT, and total SOD in heart.
65	Rat	PTU	↓ SOD and CAT and ↑ GPx in testis.
66	Rat	PTU	In brain: ↑ Total and MnSOD, CAT, total and Se-dependent GPx. ↓ Se-independent GPx, and GR.
55	Rat	MMI	In cerebral cortex: ↑ Total SOD, Cu, ZnSOD, and GPx, and ↓ MnSOD. ↔ CAT.
67	Rat	Na^131^I	↓ SOD, ↔ CAT, and ↑ GPx in kidney. ↓ Plasma GPx.
47	Rat	PTU	Cu, ZnSOD ↔ in extensor digitorum longus and soleus muscles, ↑ heart, and ↓ liver. GPx ↔ in soleus muscle and liver ↑ extensor digitorum longus muscle and heart.
68	Rat	MMI	GPx ↑ in gastrocnemius muscle and heart, and ↔ in liver. GR in ↔ heart, gastrocnemius muscle, and liver.

Ref. = reference, MHI = Method of hypothyroidism induction, PTU = 6-n-propyl-2-thiouracil, MMI = methimazole, ↑ = increase, ↓ = decrease, ↔ = without change, SOD = superoxide dismutase, MnSOD = manganese-dependent superoxide dismutase, Cu, ZnSOD = copper,zinc-dependent superoxide dismutase, Se = selenium, GPx = Glutathione peroxidase, GR = glutathione reductase, CAT = catalase.
